# Morphological Changes in Thoracic Internal Structures of *Asiophrida xanthospilota* (Coleoptera: Chrysomelidae) During Pupal Period

**DOI:** 10.3390/insects16111133

**Published:** 2025-11-05

**Authors:** Irfan Haider, Le Zong, Wenjie Li, Youyou Yang, Zulong Liang, Xinyi Zhou, Yanting Wang, Sipei Liu, Siqin Ge

**Affiliations:** 1 State Key Laboratory of Animal Biodiversity Conservation and Integrated Pest Management, Institute of Zoology, Chinese Academy of Sciences, Beijing 100101, China; irfan.ucas@yahoo.com (I.H.); zongle@ioz.ac.cn (L.Z.); liwenjie@ioz.ac.cn (W.L.); yangyouyou24@mails.ucas.ac.cn (Y.Y.); liangzl@szpu.edu.cn (Z.L.); zhouxinyi@ioz.ac.cn (X.Z.); biologyscience163@outlook.com (Y.W.); 2 University of Chinese Academy of Sciences, Beijing 100101, China; 3 Plant Protection Research Center, Shenzhen Polytechnic University, Shenzhen 518055, China

**Keywords:** flea beetle, metamorphosis, morphology, micro-CT, 3D reconstruction

## Abstract

The thorax, which serves as the primary center of locomotion for insects, consists of a highly intricate skeletomuscular system. The thoracic morphological transformations during the pupal stage reveal the developmental formation of locomotor systems. *Asiophrida xanthospilota* is not only capable of flight but is also an exceptional jumper. In this study, we employed micro-CT and 3D reconstruction to document the thoracic internal anatomy of this species on pupal days 1, 2, 4, 6, 8, 10, and 12, with the goal of achieving a more comprehensive understanding of their locomotion mechanisms. In *A. xanthospilota*, a membrane connecting the occiput and metaphragma is present from pupal days 2 to 8, serving as the attachment site for the notal muscles. Morphological changes in some endoskeletal structures during development result in corresponding shifts in the origins or insertions of associated muscles. On the first day, most muscles are present in the pro- and mesothorax, while the metathorax contains comparatively few, a pattern likely linked to the primary reliance on jumping locomotion of the species. Some muscles that appear only during the early and middle pupal stages might function in structural support. Muscular morphology undergoes diverse changes that are difficult to generalize. On day 4, the muscles show a comparatively small relative volume, which might reflect experimental error or other underlying factors. In addition, the gut and nerve remain largely unchanged. It should be noted that the stroma within the thoracic cavity might have hindered the precise identification of muscles.

## Simple Summary

The thorax is the main body region responsible for insect movement and contains a complex system of skeletons and muscles. During the pupal stage, its structure changes as the locomotor system develops. *Asiophrida xanthospilota* is capable of both flight and powerful jumping. In this study, we used micro-CT and 3D reconstruction to examine the internal thoracic structure on pupal days 1, 2, 4, 6, 8, 10, and 12, aiming to gain a clearer understanding of the locomotion mechanism of this species. We found that from days 2 to 8, a membrane connects parts of the head and thorax, providing an attachment site for muscles. Some internal skeletal changes during development also shift where muscles attach. On the first day, most muscles were already visible in the pro- and mesothorax, but fewer were in the metathorax, likely because this beetle mainly relies on jumping. Some muscles appeared only in the early and middle pupal stages and probably provided temporary support. Overall, the muscles changed in many ways, and on day 4, their volumes were unexpectedly small, which could be due to an error or other factors. The gut and nerves changed little. It should be noted that background tissue in the thorax sometimes makes it harder to identify muscles clearly.

## 1. Introduction

The thorax features a complex system of skeletons and muscles, functioning as the primary center of locomotion in insects [1]. The pterothoracic segments, comprising the meso- and metathorax, contain various direct and indirect muscles that facilitate flight movements [2,3,4]. It has also been found that certain muscles in the prothorax control neck movements, helping to stabilize the head during flight and maintain visual acuity [5,6,7]. Meanwhile, several thoracic muscles connect to the legs, particularly the coxae and trochanters, assisting in controlling leg movements such as walking, jumping, and swimming [8,9,10].

The pupal stage, as an apomorphy of holometabolous insects, constitutes a crucial developmental phase for investigating the evolution of insect metamorphosis and ecological adaptation [11,12,13,14]. Documenting the morphological transformations of the thorax in holometabolous insects during pupal development enhances our understanding of how locomotion-related skeletal and muscular systems form, offering valuable references for the design of insect-inspired microrobots. Similar to flight behavior [15,16,17], insect jumping also has a prominent research focus in the fields of biomechanics and bionics [18,19,20]. A common species in Beijing, *Asiophrida xanthospilota* (Baly, 1881) [21] is capable of both flight and remarkable jumping performance. Using advanced imaging techniques, we found that the jumping mechanism of *A. xanthospilota* operates as a catapult and proposed a preliminary concept for robotic applications [22]. In addition, our research on the molecular mechanisms of jumping in the *A. xanthospilota* showed that the expression of resilin peaks during the late pupal stage coincides with the formation of the jumping apparatus [23].

The advent of micro-CT and 3D reconstruction techniques has enabled detailed documentation of morphological changes during the development of holometabolous insects, eliminating the need for labor-intensive and technically challenging manual dissections [24,25,26,27,28,29,30]. In Coleoptera, the pupal stages of Chrysomelidae, Tenebrionidae, Coccinellidae, and Bruchidae have been documented and, together with the other developmental data, have been used to explore scientific questions, such as ecological adaptation [31,32,33,34,35]. The external morphology of the eggs, larvae, and pupae of *A. xanthospilota* was previously described using light microscopy [36]. In this study, we employed micro-CT to collect data on thoracic internal anatomy during pupal development, aiming to examine morphological changes in the thoracic endoskeleton and musculature across the pupal stage. Using micro-CT scan data, we reconstructed the thoracic endoskeleton and musculature in 3D on pupal days 1, 2, 4, 6, 8, 10, and 12, and provided detailed internal morphological descriptions. In the future, we aim to combine this study with other research on *A. xanthospilota* to develop a more comprehensive understanding of the species from a perspective of locomotion mechanisms.

## 2. Materials and Methods

**Figure 1:**
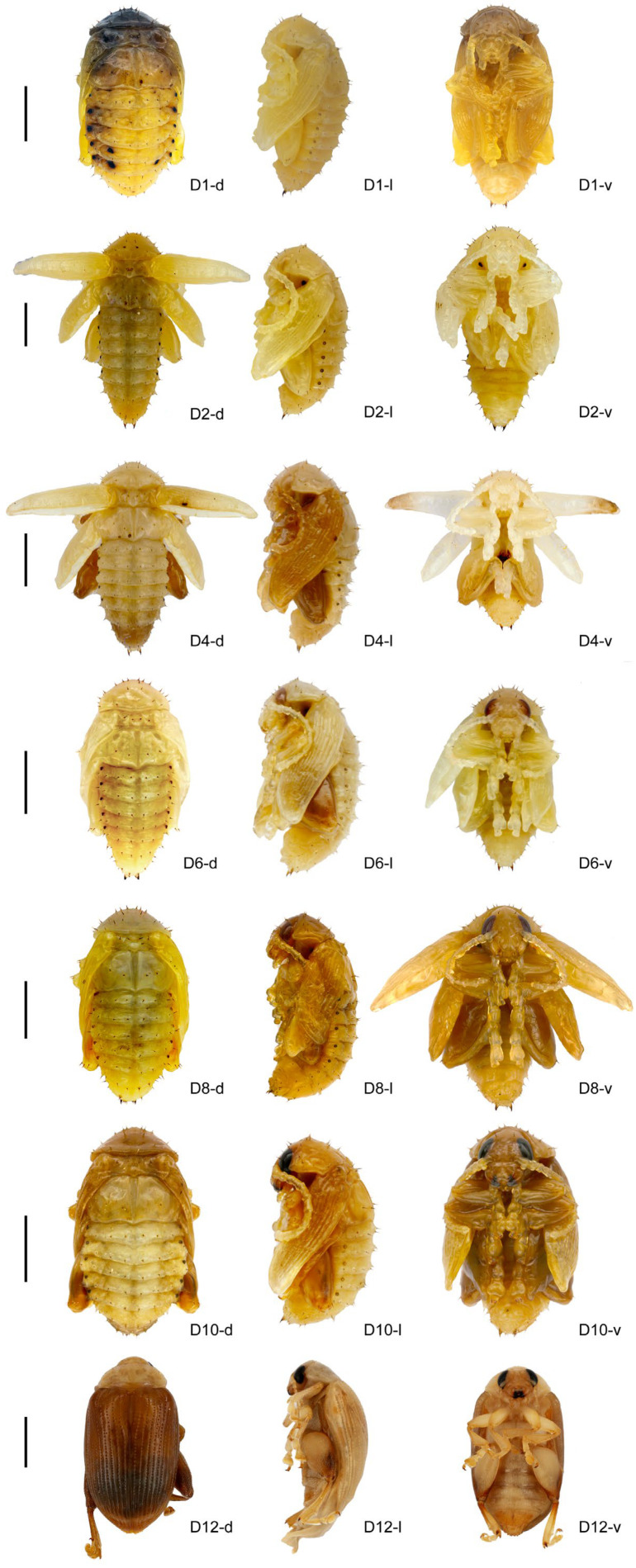
Pupal appearance of *Asiophrida xanthospilota*. (**D1-d**, **D2-d**, **D4-d**, **D6-d**, **D8-d**, **D10-d, and D12-d**) dorsal view on D1, 2, 4, 6, 8, 10, and 12; (**D1-l**, **D2-l**, **D4-l**, **D6-l**, **D8-l**, **D10-l, and D12-l**) lateral view on D1, 2, 4, 6, 8, 10, and 12; (**D1-v**, **D2-v**, **D4-v**, **D6-v**, **D8-v**, **D10-v, and D12-v**) ventral view on D1, 2, 4, 6, 8, 10, and 12. Scale bars = 2.0 mm. Each scale bar corresponds to the adjacent developmental stage images on the right.

The eggs and larvae of *Asiophrida xanthospilota* were collected either from the back mountain of the National Botanical Garden (N 40°0′40.23″, E 116°12′24.02″) or around Huangbai Temple in Yanqing District (N 40°31′32.83″, E 115°59′30.07″) in Beijing, China, from mid-to-late April to early May in 2020 to 2023. They were raised in the incubator maintained at a constant temperature of 25 °C and 55% relative humidity under a 14 h-10 h light–dark photoperiod. The pupal stage of *A. xanthospilota* lasts a total of twelve days. The developmental process was recorded daily after the onset of pupation. Specimens were collected on the 1st, 2nd, 4th, 6th, 8th, 10th, and 12th day of the pupal stage (denoted as D1, D2, D4, D6, D8, D10, and D12 in the following text) and photographed using an SLR camera (Canon 5D III, IZCAS, Beijing, China). A focus-stacking technique was applied by selecting the highest and lowest focal planes of each specimen and capturing a series of images at different depths of field. The images were subsequently combined using Helicon Focus 6.0 (Helicon Soft Ltd., Kharkov, Ukraine) to generate fully focused composite photographs (Figure 1).

The collected specimens were preserved in 75% ethanol for over one week and then sequentially dehydrated in 75%, 80%, 85%, 90%, and 95% ethanol for 20–30 min each, followed by three rounds of dehydration in 100% ethanol, each lasting 20–30 min. Specimens dehydrated with ethanol were dried using a critical point dryer (Leica EM CPD300, IZCAS, Beijing, China). If complete drying was not achieved, a freeze dryer was subsequently used to ensure thorough desiccation (ALPHA LDplus 1-2, IZCAS, Beijing, China). During micro-CT scanning (Zeiss MicroXCT-400, IZCAS, Beijing, China), the specimen was stabilized within a pipette tip using tissue paper, and the tip was fixed onto the micro-CT specimen holder. The thoracic region of each specimen was three-dimensionally reconstructed from the micro-CT image stack using Amira 6.0 (Thermo Fisher Scientific, Waltham, MA, USA). For micro-CT scanning, we used a voltage of 60 kV and a current of 133 μA, with a magnification of 3.9678 under a 4× objective. The thoracic length, width, and height were measured using the “Measure” tool in Amira, and muscle volumes were quantified with the “Material Statistics” module. Unit conversion was performed based on the scale bar in the micro-CT slices. The segmented structures were exported as TIFF file stacks into VG Studio Max 3.4 (Volume Graphics, Heidelberg, Germany) for volume rendering. The final images were edited in Adobe Photoshop 2017 (Adobe Inc., Mountain View, CA, USA) and arranged with labels in Adobe Illustrator 2017 (Adobe Inc., Mountain View, CA, USA).

Endoskeletal terminology follows Ruan et al. [37], while muscle terminology follows Fredrich and Beutel [38].

## 3. Results

The thoracic internal structures during the pupal development were described based on the observations from 3D reconstructions (Figure 2, Figure 3, Figure 4, Figure 5, Figure 6, Figure 7, Figure 8 and Figure 9).

### 3.1. Thoracic Skeletons

#### 3.1.1. D1

In the dorsal view, the length ratio of the pro-, meso-, and metathorax is 11:6:11.

**Figure 2:**
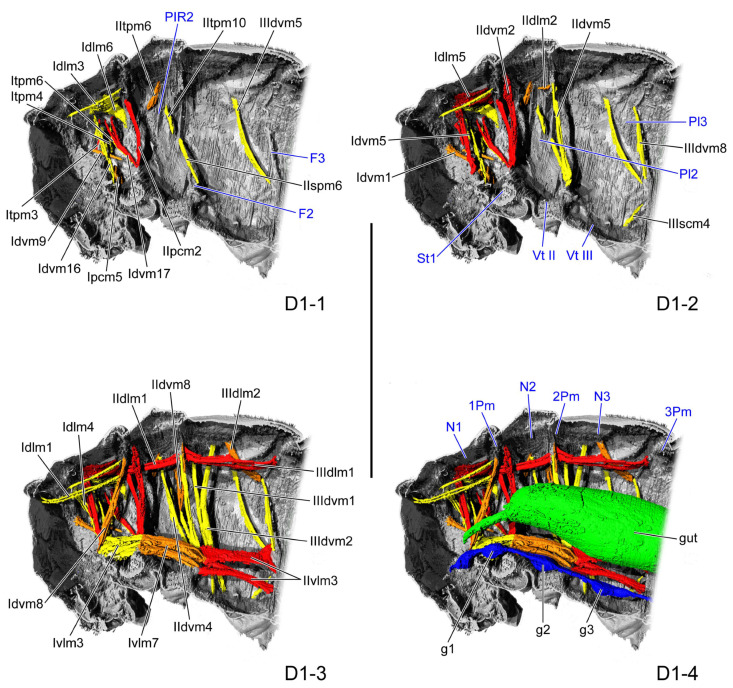
Thoracic internal structures of pupal *Asiophrida xanthospilota* on D1 in sagittal section. Skeletal structures are marked in gray and labeled in dark blue; muscles are marked in red, orange, or yellow and labeled in black; the gut is marked in green and labeled in black; and the nerve is marked in dark blue and labeled in black. (**D1-1**–**D1-4**) The numbers 1–4 indicate a gradual movement from the lateral position of the thorax to the proximal position. Scale bars = 2.0 mm. Abbreviations: 1/2/3Pm—pro-/meso-/metaphragma; F2/3—meso-/metafurca; g1/2/3—pro-/meso-/metathoracic ganglion; N1/2/3—pro-/meso-/metanotum; Pl2/3—meso-/metapleuron; PlR2—mesopleural ridge; St1—prosternum; and Vt II/III—meso-/metaventrite.

The pronotum (N1: Figure 2(D1-4)) from the lateral view appears as an irregular quadrilateral, the widest dorsally, with its anterior margin exceeding the posterior margin in length. On the inner side, a short and straight ridge extends from the midpoint of the posterior margin to the postero-median region. The narrow and short prophragma (1Pm: Figure 2(D1-4)) expands antero-ventrad from the posterior margin of the pronotum. The lateral margin of the pronotum extends dorso-posterad from the dorso-lateral margin of the occiput, connecting with the antero-dorsal edge of the mesopleuron, thus delimiting the dorsal pronotum from the ventral prosternum. The prosternum (St1: Figure 2(D1-2)) is shortest at the midline, where it invaginates dorsad to form a ridge. The posterior margin of the prosternum is also invaginated antero-dorsad to form a ridge that extends dorso-laterad from the midline to the postero-lateral corner of the pronotum. Ventrally, the prosternum projects ventrad and bulges to form the procoxa.

The mesonotum (N2: Figure 2(D1-4)) in the lateral view appears trapezoidal, with parallel anterior and posterior margins, and the anterior is shorter than the posterior. The lateral margin bends dorso-posterad and extends from the postero-lateral margin of the pronotum to the antero-lateral corner of the metanotum. The postero-lateral margin expands ventrad to form a narrow, elongated triangular mesophragma (2Pm: Figure 2(D1-4)) tapering proximad. Beneath the mesonotum is the elongated mesopleuron (Pl2: Figure 2(D1-2)), nearly parallelogram-shaped, with its anterior and posterior margins longer than the dorsal and ventral margins. The vertical mesopleural ridge (PlR2: Figure 2(D1-1)) extends ventrad from the postero-median dorsal margin to the central region. The mesoventrite (Vt II: Figure 2(D1-2)) is also shortest at the median area along the midline. The bulged mesocoxa projects ventrad from the mesoventrite, with its proximal and posterior margins invaginating dorsad to form a bent postero-proximal ridge, while its posterior coxal margin extends latero-dorsad to connect with the postero-ventral margin of the mesopleuron. The mesofurca (F2: Figure 2(D1-1)) is a slightly raised triangular process located on the proximal point of the posterior coxal margin, with its lateral margin expanding in an antero-posterior direction.

The metanotum (N3: Figure 2(D1-4)) in the lateral view also exhibits a trapezoidal form, with parallel anterior and posterior margins, the anterior shorter than the posterior. Internally, the metanotum has a straight median ridge positioned along the midline, between the midpoints of the anterior and posterior margins. The posterior margin of the metanotum extends slightly antero-ventrad to form a narrow metaphragma (3Pm: Figure 2(D1-4)). The metapleuron (Pl3: Figure 2(D1-2)) occupies a large area beneath the metanotum, fused with the metaventrite and lacking a clear boundary. Between the paired metapleurals, the metaventrite (Vt III: Figure 2(D1-2)) occupies a large area, with its wide median region invaginated dorsad. The extremely bulged metacoxa arises from the postero-ventral region of the metathorax. The metafurca (F3: Figure 2(D1-1)) is a long and slender process with a tapering apex, extending latero-dorsad from the median area of the metacoxal anterior margin toward the postero-median margin of the metapleuron.

#### 3.1.2. D2

**Figure 3:**
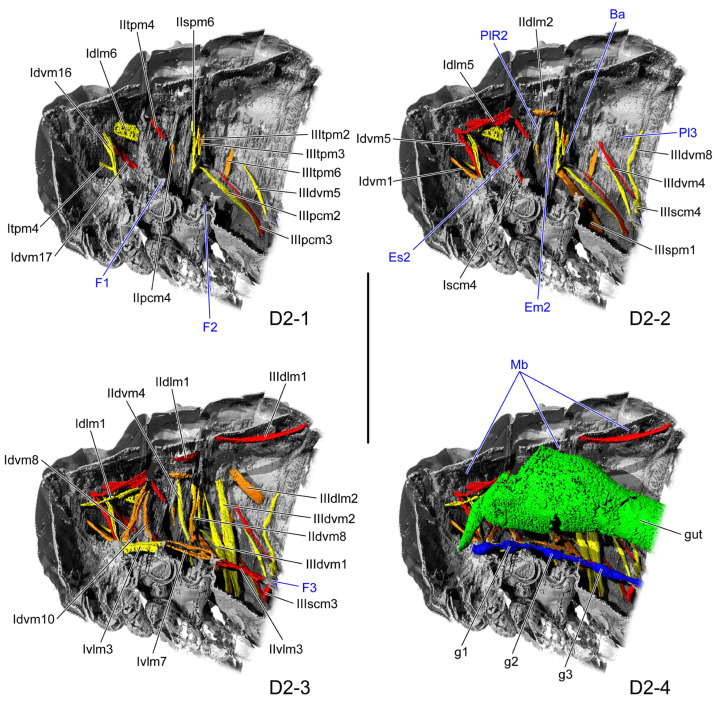
Thoracic internal structures of pupal *Asiophrida xanthospilota* on D2 in sagittal section. Skeletal structures are marked in gray and labeled in dark blue; muscles are marked in red, orange, or yellow and labeled in black; the gut is marked in green and labeled in black; and the nerve is marked in dark blue and labeled in black. (**D2-1**–**D2-4**) The numbers 1–4 indicate a gradual movement from the lateral position of the thorax to the proximal position. Scale bars = 2.0 mm. Abbreviations: Ba—metabasalar sclerite; Em2—mesepimeron; Es2—mesanepisternum; F1/2/3—pro-/meso-/metafurca; g1/2/3—pro-/meso-/metathoracic ganglion; Mb—membrane; Pl3—metapleuron; and PlR2—mesopleural ridge.

In the dorsal view, the length ratio of the pro-, meso-, and metathorax is 15:8:14. Beneath the thoracic notum, a membrane (Mb: Figure 3(D2-4)) beneath the thoracic notum extends between the occiput and the metaphragma, connecting medially with both the pro- and mesophragma, while its lateral margins are continuous with those of the notum. All muscles associated with the notum are positioned beneath this membrane. The midline of the prosternal posterior ridge is concave anterad. From its midpoint toward the lateral margin arises a rectangular process extending dorsad, and there is the profurca (F1: Figure 3(D2-1)), whose dorso-laterad corner is thickened. The mesopleural ridge (PlR2: Figure 3(D2-2)) extends antero-ventrad from the midpoint of the dorsal margin to the antero-ventral corner of the mesopleuron, medially bearing a ventrad-projecting process. Accordingly, the mesopleuron is divided into the anterior elongated inverted-triangular mesanepisternum (Es2: Figure 3(D2-2)) and the posterior slightly postero-ventrad bent elongated rectangular mesepimeron (Em2: Figure 3(D2-2)). The mesofurca is divided into anterior and posterior parts. The proximal 2/3 of the anterior part extends latero-dorsad, and then bends at the distal 1/3, where they terminate in an acute apex. The posterior part extends latero-dorsad from the midline, attaching to the anterior part, and reaches the lateral 1/3 curvature of the latter, where it terminates in an enlarged dorsad-projecting end. The antero-dorsal margin of the metapleuron expands proximad and concaves anterad to form the metabasalar sclerite (Ba: Figure 3(D2-2)). The metafurca (F3: Figure 3(D2-3)) is positioned medially along the posterior margin of the metaventrite and bears a broad stem. From the dorso-lateral margin of the stem, the stout metafurcal arm extends antero-laterad and terminates in an expanded apex.

#### 3.1.3. D4

**Figure 4:**
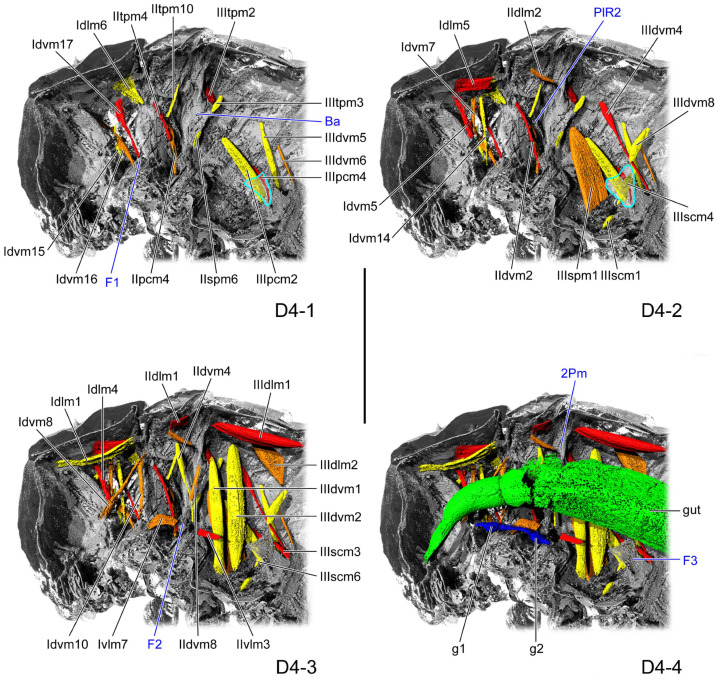
Thoracic internal structures of pupal *Asiophrida xanthospilota* on D4 in sagittal section. Skeletal structures are marked in gray and labeled in dark blue; muscles are marked in red, orange, or yellow and labeled in black; the gut is marked in green and labeled in black; and the nerve is marked in dark blue and labeled in black. Skeletal elements circled by a light blue line are rendered transparent to show the muscles behind them. (**D4-1**–**D4-4**) The numbers 1–4 indicate a gradual movement from the lateral position of the thorax to the proximal position. Scale bars = 2.0 mm. Abbreviations: 2Pm—mesophragma; Ba—metabasalar sclerite; F1/2/3—pro-/meso-/metafurca; g1/2—pro-/mesothoracic ganglion; and PlR2—mesopleural ridge.

In the dorsal view, the length ratio of the pro-, meso-, and metathorax is 6:3:5. The profurca (F1: Figure 4(D4-1)) is situated between the prosternal midline and the procoxa, forming an enlarged base that bears a slightly protuberant apical process. The narrow mesophragma (2 Pm: Figure 4(D4-4)) expands ventrad from the mesonotal posterior margin, bearing a tapering process that extends latero-ventrad from its latero-ventral margin. The dorsal portion of the mesopleural ridge (PlR2: Figure 4(D4-2)) projects proximad, forming an elongated triangular structure. The stout mesofurcal arm (F2: Figure 4(D4-3)) extends dorso-laterad from the postero-proximal margin of the mesocoxa, terminating in an obtuse apex. From its antero-proximal margin arises a broad process that projects slightly antero-dorsad. The metaphragma extends ventrad from the metanotal posterior margin. The antero-dorsal margin of the metapleuron expands proximad to form the metabasalar sclerite (Ba: Figure 4(D4-1)). The broad stem of the metafurca (F3: Figure 4(D4-4)) extends antero-dorsad from the median margin of the metaventrite. Anteriorly, it expands into a flat triangular plate bearing an obtuse anterior apex and paired acute lateral processes.

**Figure 5:**
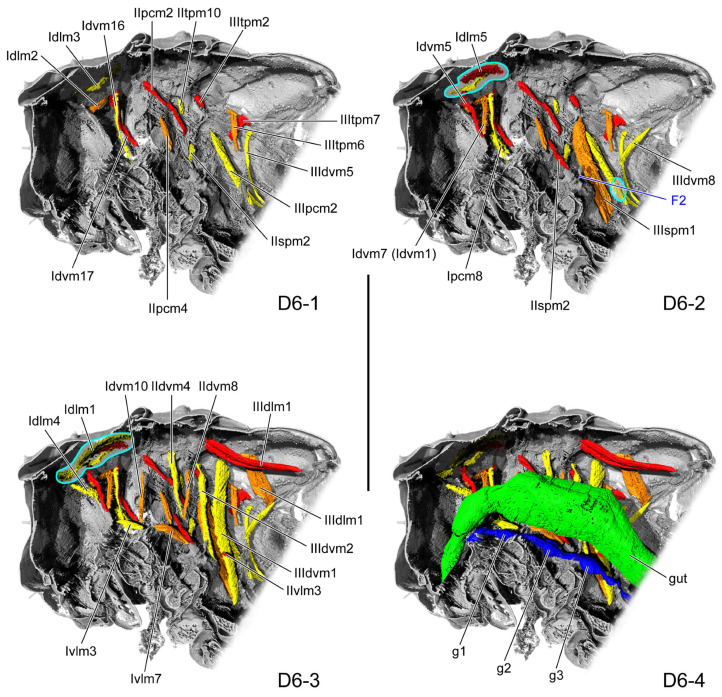
Thoracic internal structures of pupal *Asiophrida xanthospilota* on D6 in sagittal section. Skeletal structures are marked in gray and labeled in dark blue; muscles are marked in red, orange, or yellow and labeled in black; the gut is marked in green and labeled in black; and the nerve is marked in dark blue and labeled in black. Skeletal elements circled by light blue lines are rendered transparent to show the muscles behind them. (**D6-1**–**D6-4**) The numbers 1–4 indicate a gradual movement from the lateral position of the thorax to the proximal position. Scale bars = 2.0 mm. Abbreviations: F2—mesofurca; and g1/2/3—pro-/meso-/metathoracic ganglion.

**Figure 6:**
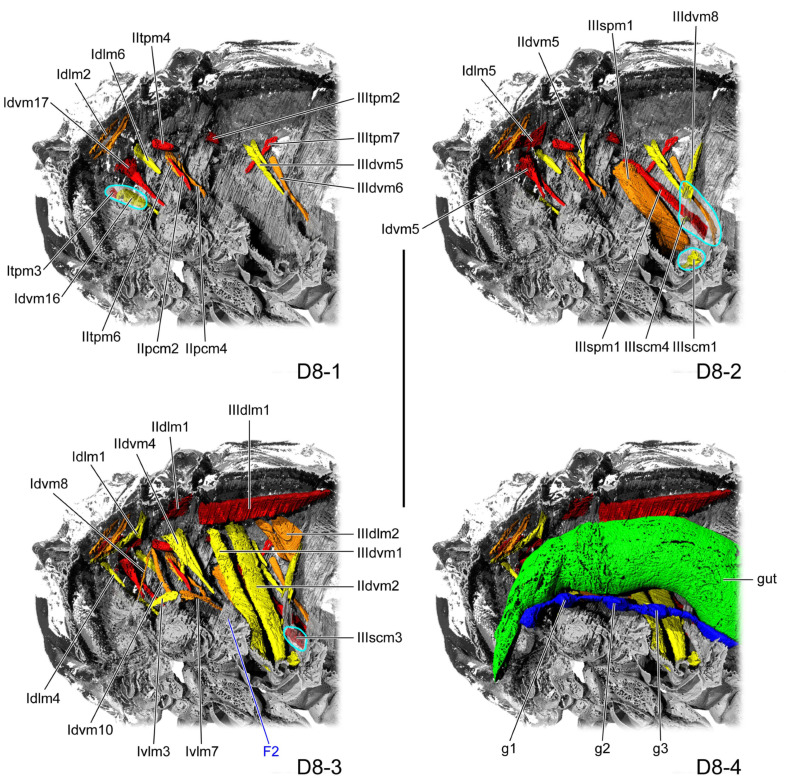
Thoracic internal structures of pupal *Asiophrida xanthospilota* on D8 in sagittal section. Skeletal structures are marked in gray and labeled in dark blue; muscles are marked in red, orange, or yellow and labeled in black; the gut is marked in green and labeled in black; and the nerve is marked in dark blue and labeled in black. Skeletal elements circled by light blue lines are rendered transparent to show the muscles behind them. (**D8-1**–**D8-4**) The numbers 1–4 indicate a gradual movement from the lateral position of the thorax to the proximal position. Scale bars = 2.0 mm. Abbreviations: F2—mesofurca; and g1/2/3—pro-/meso-/metathoracic ganglion.

#### 3.1.4. D6

In the dorsal view, the length ratio of the pro-, meso-, and metathorax is 18:7:16. The mesophragma gradually broadens laterad along the mesonotal posterior margin, giving rise from its latero-ventral margin to an obtuse process that extends proximo-ventrad. The broad short stem of the mesofurca (F2: Figure 5(D6-2)) arises from the median portion of the posterior margin of the mesoventrite. From it extend paired processes directed laterad, each terminating in a tapering apex. The basal stem of the metafurca is narrow, and its slender arm bends postero-ventrad.

#### 3.1.5. D8

In the dorsal view, the length ratio of the pro-, meso-, and metathorax is 14:5:14. The mesofurca (F2: Figure 6(D8-3)) divides at the midline, with each side extending antero-dorsad from the median portion of the anterior margin of the bulged mesocoxa and forming a triangular process that tapers to an acute apex.

**Figure 7:**
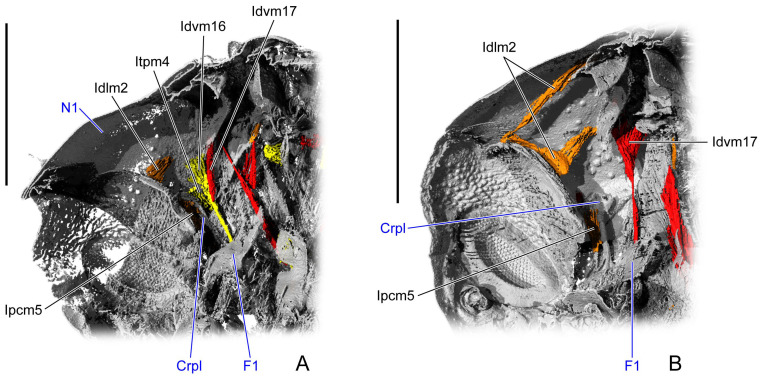
Prothoracic internal structures of pupal *Asiophrida xanthospilota*. (**A**) D10, lateral view; (**B**) D12, latero-rear view. Skeletal structures are marked in gray and labeled in dark blue; muscles are marked in red, orange, or yellow and labeled in black. Scale bars = 1.0 mm. Left scale bar: (**A**); right scale bar: (**B**). Abbreviations: Crpl—crytopleuron; F1—profurca; and N1—pronotum.

#### 3.1.6. D10

**Figure 8:**
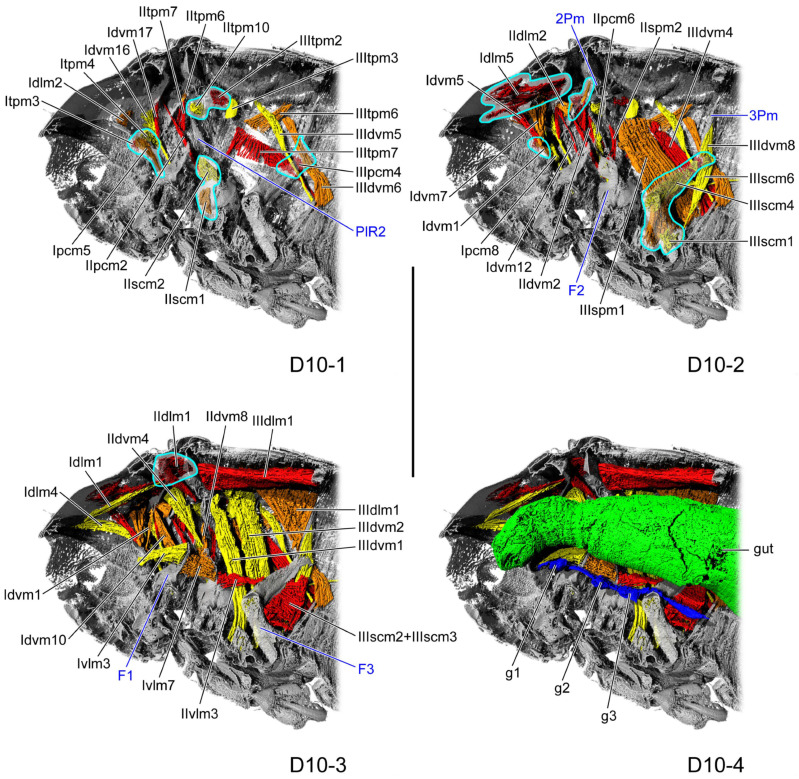
Thoracic internal structures of pupal *Asiophrida xanthospilota* on D10 in sagittal section. Skeletal structures are marked in gray and labeled in dark blue; muscles are marked in red, orange, or yellow and labeled in black; the gut is marked in green and labeled in black; and the nerve is marked in dark blue and labeled in black. Skeletal elements circled by light blue lines are rendered transparent to show the muscles behind them. (**D10-1**–**D10-4**) The numbers 1–4 indicate a gradual movement from the lateral position of the thorax to the proximal position. Scale bars = 2.0 mm. Abbreviations: 2/3Pm—meso-/metaphragma; F1/2/3—pro-/meso-/mesofurca; g1/2/3—pro-/meso-/metathoracic ganglion; and PlR2—mesopleural ridge.

In the dorsal view, the length ratio of the pro-, meso-, and metathorax is 16:5:14. The membrane beneath the thoracic notum is absent. The median region of the prophragma is concave posterad, whereas the lateral regions project anterad. The slender crytopleuron (Crpl: Figure 7A) extends antero-dorsad from the lateral margin of the procoxal rim, its apex slightly enlarged and bent dorso-posterad near the dorso-lateral area of the pronotum. The broad profurcal arm (F1: Figure 8(D10-3)) arises from the dorso-median surface of the bulged profurcal base, expanding dorso-posterad and curving postero-ventrad. The mesophragma (2Pm: Figure 8(D10-2)) is broad medially and tapers laterally, bearing from its latero-ventral margin a process that extends latero-ventrad and bends proximo-ventrad. The mesopleural ridge (PlR2: Figure 8(D10-1)) bends and expands postero-dorsad. The mesofurcal arm (F2: Figure 8(D10-2)) has a bulged base, with its distal portion slender, slightly curved, and projecting antero-laterad. The process arises from the ventro-lateral margin of the metaphragma (3Pm: Figure 8(D10-2)), tapering gradually towards its distal end. The stout metafurcal arm (F3: Figure 8(D10-3)) extends postero-dorsad, tapering distally and approaching the distal end of the ventro-lateral process of the metaphragma.

**Figure 9:**
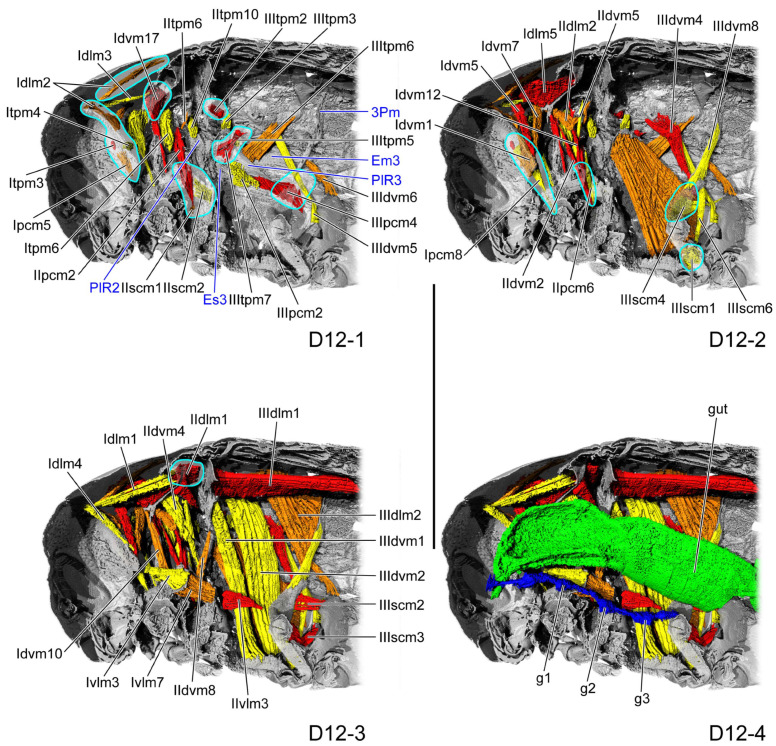
Thoracic internal structures of pupal *Asiophrida xanthospilota* on D12 in sagittal section. Skeletal structures are marked in gray and labeled in dark blue; muscles are marked in red, orange, or yellow and labeled in black; the gut is marked in green and labeled in black; and the nerve is marked in dark blue and labeled in black. Skeletal elements circled by light blue lines are rendered transparent to show the muscles behind them. (**D12-1**–**D12-4**) The numbers 1–4 indicate a gradual movement from the lateral position of the thorax to the proximal position. Scale bars = 2.0 mm. Abbreviations: 3Pm—metaphragma; Em3—metepimeron; Es3—metanepisternum; g1/2/3—pro-/meso-/metathoracic ganglion; and PlR2/3—meso-/metapleural ridge.

#### 3.1.7. D12

In the dorsal view, the pronotum entirely covers the mesonotum. The length ratio of the pro- and metathorax is 1:1. A process extends latero-ventrad from the ventro-lateral corner of the prophragma, gradually tapering toward its distal end. The distal end of the crytopleuron (Crpl: Figure 7B) expands into a disk-like structure. The mesopleural ridge (PlR2: Figure 9(D12-1)) is narrow, its ventral end extending to the posterior portion of the mesopleural ventral margin. As a result, the mesanepisternum forms a broader triangle with a broad ventral region, larger in than the posterior narrow elongated mesepimeron. The ventro-lateral process of the metaphragma (3Pm: Figure 9(D12-1)) is slender. The metapleural ridge (PlR3: Figure 9(D12-1)) extends from the metabasalar sclerite to the metapleural postero-ventral corner, with its anterior portion folded dorsad, separating the metapleuron into the ventro-anterior metanepisternum (Es3: Figure 9(D12-1)) and the dorso-posterior metepimeron (Em3: Figure 9(D12-1)).

#### 3.1.8. Measurement

For each developmental stage, we measured the following distances in the micro-CT image stacks to represent length, width, and height. Length is defined as the distance from the midpoint of the pronotal anterior margin to the metanotal posterior margin. Width is defined as the distance between the hindwings. Height is defined as the vertical extent of the metanotal posterior margin in lateral view. The product of these three measurements was used to approximate the volume. Each value was measured three times. The products were calculated individually, and the average of these products was then obtained (Table 1).

### 3.2. Thoracic Muscles

By combining the muscles from all pupal developmental stages, we identified a total of 65 muscles, including 25 in the prothorax, 18 in the mesothorax, and 22 in the metathorax. Table 2 records the presence or absence of thoracic muscles and their numbers at each developmental stage. Each muscle at each stage during pupal development is described in detail. The origin, insertion, planar shape, and direction of curvature of the muscle at its earliest appearance were recorded. Any changes in subsequent stages were recorded again, while unchanged muscles were not re-recorded. We also measured the volume of each muscle (Table 3 and Table 4). The absolute volume was calculated based on the scale bars in the micro-CT results. For relative volume, the muscular absolute volume was compared with the product of thoracic length, width, and height.

#### 3.2.1. Prothoracic Muscles

- *Idlm1 M. prophragma-occipitalis*

D1: O (= origin): ventro-lateral margin of prophragma. I (= insertion): dorso-lateral margin of occiput. Approximate parallelogram, original and insertional ends equal, twisted, and bent ventrad at anterior 1/5.

D2: O: suspended beneath prophragma. I: dorso-lateral margin of occiput. Curved.

D4: O: ventro-lateral margin of prophragma. I: dorso-median margin of occiput. Elongated trapezoid, original end narrower than insertional end, bent ventrad.

D6: O: dorso-lateral area of the anterior face of the phragma. I: dorso-median margin of occiput. Approximate rectangle, expanded original end broader than insertional end, curved.

D8: Elongated triangle, original end narrower than expanded insertional end, slightly bent ventrad.

D10: O: ventro-median margin of prophragma. I: dorso-median margin of occiput. Broad medially and narrowing towards both ends, straight.

D12: O: ventro-median area of anterior face of prophragma. I: dorso-median margin of occiput. Elongated triangle, narrowing towards occiput, straight.

- *Idlm2 M. pronoto-occipitalis*

D1, 2, and 4: Absent.

D6: O: latero-median area of pronotum. I: dorso-lateral margin of occiput. Elongate triangle, narrowing towards the pronotum, straight.

D8: Two bundles, proximal one shorter than distal one. O: postero-median area of pronotum. I: dorso-lateral margin of occiput. Broad medially and narrowing towards both ends. Proximal bundle straight, and distal bundle bent ventro-laterad at anterior 1/4.

D10: O: latero-median area of pronotum. I: dorso-lateral margin of occiput. Triangle, narrowing towards the pronotum, slightly bent antero-dorsad.

D12: Two bundles, proximal one longer than distal one. Proximal bundle: O: postero-median area of pronotum. I: dorso-lateral margin of occiput. Elongate triangle, narrowing towards occiput, slightly bent at the posterior 1/3. Distal bundle: O: latero-median area of pronotum. I: dorso-lateral margin of occiput. Elongated triangle, narrowing towards pronotum, insertional end extends proximad to connect with insertional end of proximal bundle, slightly bent antero-dorsad.

- *Idlm3 M. prophragma-cervicalis*

D1: O: ventro-median margin of prophragma. I: dorso-median area of pronotum. Broad medially and narrowing towards both ends, slightly bent ventrad.

D2 and 4: Absent.

D6: O: postero-median area of pronotum. I: antero-lateral area of pronotum. Approximate elongated triangle, narrowing towards anterior area of pronotum, bent ventrad.

D8 and 10: Absent.

D12: O: dorso-lateral area of prophragma. I: antero-lateral area of pronotum. Elongated triangle, narrowing towards prophragma, slightly bent ventrad.

- *Idlm4 M. cervico-occipitalis dorsalis*

D1: O: antero-lateral area of pronotum. I: ventro-lateral margin of occiput. Elongated triangle, narrowing towards occiput, curved.

D2: Absent.

D4: O: antero-lateral area of pronotum. I: dorso-lateral margin of occiput. Straight.

D6: O: suspended beneath dorso-lateral margin of occiput. I: ventro-lateral margin of occiput.

D8: O: antero-lateral area of pronotum. I: meso-lateral margin of occiput.

D10: O: antero-lateral area of pronotum. I: ventro-lateral margin of occiput. Slightly bent postero-laterad.

D12: Almost the same as that on D10.

- *Idlm5 M. pronoto-phragmalis anterior*

D1: O: meso-lateral area of pronotum. I: ventro-lateral area of anterior face of prophragma. Approximate trapezoid, insertional end broader than original end, slightly bent ventrad.

D2: O: antero-lateral area of pronotum. I: suspended beneath prophragma. Approximate triangle, narrowing towards pronotum, straight.

D4: O: meso-lateral area of pronotum. I: ventro-lateral area of anterior face of prophragma. Parallelogram, straight.

D6: O: antero-lateral area of pronotum. I: lateral area of anterior face of prophragma. Bent dorso-posterad.

D8: O: antero-lateral area of pronotum. I: lateral area of anterior face of prophragma. Elongated triangle, narrowing towards pronotum, straight.

D10: O: antero-median area of pronotum. I: latero-median area of anterior face of prophragma. Broad and flat, straight.

D12: Broad and flat, insertional end extends proximad toward the midline, straight.

- *Idlm6 M. pronoto-phragmalis posterior*

D1: O: postero-lateral area of pronotum. I: dorso-lateral area of the anterior face of prophragma. Flat triangle, narrowing towards the pronotum, bent dorso-posterad.

D2: Flat rectangle, bent ventro-proximad.

D4: Flat triangle, narrowing towards the pronotum, straight.

D6: Absent.

D8: O: postero-lateral area of pronotum. I: ventro-lateral margin of prophragma. Elongated triangle, narrowing towards prophragma, straight.

D10 and 12: Absent.

- *Idvm1 M. cervico-occipitalis anterior*

D1: O: ventro-lateral margin of occiput. I: lateral margin of occiput. Elongated rectangle, bent dorso-posterad.

D2: Broad medially and narrowing towards both ends, straight.

D4: Absent.

D6: Elongated triangle, narrowing towards the lateral area of the occiput, bent posterad.

D8: Absent.

D10: O: ventro-lateral margin of occiput. I: dorso-lateral margin of occiput. Elongated triangle, narrowing towards the dorsal area of the occiput, bent posterad.

D12: Almost the same as that on D10.

- *Idvm5 M. pronoto-cervicalis anterior*

D1: O: meso-lateral area of pronotum. I: ventro-lateral margin of occiput. Approximate elongated triangle, narrowing towards occiput, curved.

D2: Bent posterad.

D4: Almost the same as that on D2.

D6: Curved.

D8: Straight.

D10: Approximate rectangle.

D12: Elongated triangle, narrowing towards occiput.

- *Idvm7 M. pronoto-cervicalis posterior*

D1 and 2: Absent.

D4: O: postero-lateral area of pronotum. I: ventro-lateral margin of occiput. Flat trapezoid, both original and insertional ends equal, straight.

D6: Approximate rectangle, bent postero-ventrad.

D8: Absent.

D10: Elongated triangle, narrowing towards the occiput, straight.

D12: Almost the same as that on D10.

- *Idvm8 M. prophragma-tentorialis*

D1: Two bundles, cross medially. O: ventro-median margin of prophragma. I: venrto-lateral margin of the occiput. Broad medially and narrowing towards both ends, straight.

D2: O: beneath prophragma. I: ventro-lateral margin of the occiput. Rectangle, slightly curved.

D4: O: ventro-median margin of prophragma. I: venrto-lateral margin of the occiput. Elongated triangle, narrowing towards prophragma, straight.

D6: Absent.

D8: Broad medially and narrowing towards both ends, straight.

D10: Elongated triangle, narrowing towards occiput, straight.

D12: Absent.

- *Idvm9 M. profurca-occipitalis*

D1: O: extending towards ventral portion of intersegmental ridge between pro- and mesopleuron. I: extending towards lateral margin of occiput. Broad medially and narrowing towards both ends, straight.

D2, 4, 6, 8, 10, and 12: Absent.

- *Idvm10 M. profurca-phragmalis*

D1: Absent.

D2: O: dorso-anterior corner of profurcal arm. I: suspended beneath prophragma. Broad medially and narrowing towards both ends, bent anterad.

D4: O: dorsal area of profurcal arm. I: suspended beneath prophragma. Parallelogram, straight.

D6: O: suspended above profurcal arm. I: suspended beneath prophragma. Broad medially and narrowing towards both ends, straight.

D8: O: anterior area of profurcal arm. I: ventro-lateral margin of prophragma. Slightly bent antero-laterad.

D10: O: lateral margin of profurcal arm. I: ventro-lateral margin of prophragma. Elongated triangle, narrowing towards prophragma, straight.

D12: Curved.

- *Idvm12 M. profurca-mesonotalis*

D1, 2, 4, 6, and 8: Absent.

D10: O: latero-dorsal margin of profurcal arm. I: postero-lateral area of pronotum. Elongated triangle, narrowing towards profurca, curved.

D12: Almost the same as that on D10.

- *Idvm14 M. pronoto-trochantinalis posterior*

D1 and 2: Absent.

D4: O: postero-lateral area of pronotum. I: antero-proximal margin of procoxal rim. Elongated triangle, narrowing towards procoxa, slightly bent postero-laterad.

D6, 8, 10, and 12: Absent.

- *Idvm15 M. pronoto-trochantinocoxalis*

D1 and 2: Absent.

D4: O: antero-lateral area of pronotum. I: proximal margin of procoxal rim. Original end divides into two branches, slightly bent postero-proximad.

D6, 8, 10, and 12: Absent.

- *Idvm16 M. pronoto-coxalis anterior*

D1: O: meso-lateral area of pronotum. I: anterior margin of the procoxal rim. Original end divides into two branches, slightly curved.

D2: Two bundles, interconnected medially. Slightly bent postero-dorsad.

D4: O: suspended beneath pronotum. I: antero-lateral margin of procoxal rim. Slightly curved.

D6: O: suspended beneath the pronotum. I: anterior margin of the procoxal rim. Bent antero-dorsad.

D8: O: meso-lateral area of pronotum. I: antero-proximal margin of procoxal rim. Elongated triangle, narrowing towards procoxa, slightly bent postero-proximad.

D10: O: meso-lateral area of pronotum. I: postero-lateral margin of procoxal rim. Slightly bent antero-proximad.

D12: Straight.

- *Idvm17 M. pronoto-coxalis posterior*

D1: O: postero-lateral area of pronotum. I: postero-lateral margin of procoxal rim. Original end divides into two branches, slightly curved.

D2: Slightly bent antero-ventrad.

D4: Straight.

D6: Bent anterad.

D8: Slightly bent antero-proximad.

D10: Slightly bent antero-dorsad.

D12: Straight.

- *Itpm3 M. pronoto-pleualis anterior*

D1: O: meso-lateral area of pronotum. I: suspended beneath the pronotum. Elongated triangle, narrowing towards the pronotum, slightly bent antero-proximad.

D2, 4, and 6: Absent.

D8: Triangle, narrowing towards the pronotum, straight.

D10: O: meso-lateral area of pronotum. I: antero-dorsal area of crytopleuron. Approximate parallelogram, straight.

D12: Triangle, narrowing towards crytopleuron, straight.

- *Itpm4 M. pronoto-apodemalis anterior*

D1: O: meso-lateral area of pronotum. I: suspended beneath the pronotum. Elongated triangle, narrowing ventrad, slightly bent postero-dorsad.

D2: O: antero-lateral area of pronotum. I: suspended beneath the pronotum. Straight.

D4, 6, and 8: Absent.

D10: O: meso-lateral area of pronotum. I: postero-dorsal area of crytopleuron. Triangle, narrowing towards crytopleuron, straight.

D12: Parallelogram, straight.

- *Itpm6 M. pronoto-intersegmentalis*

D1: O: postero-lateral area of pronotum. I: postero-lateral margin of procoxal rim. Broad medially and narrowing towards both ends, bent antero-proximad.

D2, 4, 6, 8, and 10: Absent.

D12: O: postero-lateral area of pronotum. I: lateral portion of the intersegmental ridge between pro- and mesothorax. Elongated triangle, narrowing towards pronotum, straight.

- *Ipcm5 M. propleuro-coxalis inferior*

D1: O: suspended beneath the pronotum. I: antero-lateral margin of procoxal rim. Broad medially and narrowing towards both ends, straight.

D2, 4, 6, and 8: Absent.

D10: O: ventral area of the dorsal part of crytopleuron. I: lateral margin of procoxal rim. Elongated triangle, narrowing towards procoxa, slightly bent posterad.

D12: O: ventral area of the dorsal part of crytopleuron. I: suspended above procoxa. Straight.

- *Ipcm8 M. propleuro-trochanteralis*

D1, 2, and 4: Absent.

D6: O: meso-lateral area of pronotum. I: extends towards procoxa. Triangle, narrowing towards procoxa, bent antero-dorsad.

D8: Absent.

D10: O: ventral area of the dorsal part of crytopleuron. I: protrochanter. Elongated triangle, narrowing towards protrochanter, slightly bent postero-dorsad.

D12: Almost the same as that on D10.

- *Ivlm3 M. profurca-tentorialis*

D1: O: lateral portion of prosternal posterior ridge. I: ventro-lateral margin of occiput. Broad and flat, straight.

D2: O: anterior area of the profurcal arm. I: ventro-lateral margin of occiput. Rectangle, slightly curved.

D4: Absent.

D6: Broad medially and narrowing towards both ends, straight.

D8: Almost the same as that on D6.

D10: Broad and flat, straight.

D12: Approximate triangle, narrowing towards occiput.

- *Ivlm7 M. profurca-mesofurcalis*

D1: Two bundles, equal length. O: lateral portion of posterior prosternal ridge. I: antero-lateral corner of mesofurcal arm. Proximal bundle: elongated triangle, narrowing towards mesofurca, slightly bent dorsad. Distal bundle: elongated trapezoid, original end shorter than insertional end, slightly bent dorsad.

D2: Two bundles, the proximal one shorter than the distal one. O: posterior area of the profurcal arm. I: dorsal area of mesofurcal arm. Broad medially and narrowing towards both ends, straight.

D4: O: postero-ventral margin of mesofurcal arm. I: antero-proximal corner of mesofurcal arm. Trapezoid, original end broader than insertional end, slightly bent dorsad.

D6: Broad medially and narrowing towards both ends, slightly bent dorsad on anterior 1/4.

D8: Elongated trapezoid, original end broader than insertional end, straight.

D10: O: dorsal area of posterior face of the profurcal arm. I: anterior face of mesofurcal arm. Broad and flat, straight.

D12: Almost the same as that on D10.

- *Iscm4 M. profurca-coxalis lateralis*

D1: Absent.

D2: O: lateral area of the profurcal arm. I: postero-lateral margin of procoxal rim. Parallelogram, slightly bent antero-ventrad.

D4, 6, 8, 10, and 12: Absent.

#### 3.2.2. Mesothoracic Muscles

- *IIdlm1 M. prophragma-mesophragmalis*

D1: O: ventro-lateral margin of prophragma. I: ventro-lateral area of the anterior face of mesophragma. Approximate parallelogram, slightly bent ventrad.

D2: O: antero-median area of mesonotum. I: meso-lateral area of the anterior face of mesophragma. Broad medially and narrowing towards both ends, slightly bent ventrad.

D4: O: antero-median area of mesonotum. I: meso-ventral margin of mesophragma. Parallelogram, straight.

D6: Absent.

D8: O: antero-median area of mesonotum. I: meso-dorsal area of the anterior face of mesophragma.

D10: Two bundles. Dorsal bundle: O: meso-dorsal area of the posterior face of prophragma. I: dorso-lateral area of the anterior face of mesophragma. Approximate trapezoid, original end broader than insertional end, straight. Ventral bundle: O: meso-dorsal area of the posterior face of prophragma. I: latero-ventral margin of mesophragma. Elongated triangle, narrowing towards mesophragma, straight.

D12: Almost the same as that on D10.

- *IIdlm2 M. mesonoto-phragmalis*

D1: O: postero-lateral area of mesonotum. I: ventro-lateral margin of mesophragma. Elongated triangle, narrowing towards anterad, straight.

D2: O: antero-lateral area of mesonotum. I: latero-median area of the anterior face of mesophragma. Broad medially and narrowing towards both ends, straight.

D4: Elongated triangle, narrowing towards mesophragma, slightly bent ventrad.

D6 and 8: Absent.

D10: O: lateral margin of prophragma. I: lateral margin of mesophragma. Trapezoid, original end narrower than insertional end, straight.

D12: Approximate triangle, narrowing towards prophragma, insertional ends extends dorso-posterad, slightly bent proximad.

- *IIdvm2 M. mesonoto-trochantinalis anterior*

D1: O: antero-lateral area of mesonotum. I: lateral portion of the prosternal posterior ridge. Broad medially and narrowing towards both ends, bent posterad.

D2: Absent.

D4: O: suspended beneath mesonotum. I: suspended above the lateral margin of mesocoxal rim. Slightly bent postero-laterad.

D6 and 8: Absent.

D10: O: antero-lateral area of mesonotum. I: antero-lateral margin of mesocoxal rim. Elongated triangle, narrowing towards mesocoxa, bent posterad at ventral 1/3.

D12: Broad medially and narrowing towards both ends, slightly bent laterad.

- *IIdvm4 M. mesonoto-coxalis anterior*

D1: O: latero-median area of mesonotum. I: lateral margin of mesocoxal rim. Broad medially and narrowing towards both ends, slightly bent ventro-laterad.

D2: O: latero-posterior area of mesonotum. I: postero-lateral margin of mesocoxal rim. Broad medially and narrowing towards both ends, curved.

D4: Broad medially and narrowing towards both ends, bent postero-laterad.

D6: Elongated triangle, narrowing towards mesocoxa, straight.

D8: Almost the same as that on D6.

D10: O: antero-lateral area of mesonotum. I: postero-lateral margin of mesocoxal rim.

D12: Almost the same as that on D10.

- *IIdvm5 M. mesonoto-coxalis posterior*

D1: O: ventro-lateral margin of mesophragma. I: postero-lateral margin of mesocoxal rim. Broad medially and narrowing towards both ends, slightly bent antero-laterad.

D2, 4, and 6: Absent.

D8: O: postero-lateral area of mesonotum. I: ventro-median area of mesopleuron. Elongated triangle, narrowing towards the mesonotum, straight.

D10: Absent.

D12: Slightly bent proximad.

- *IIdvm8 M. mesofurca-phragmalis*

D1: O: ventro-lateral ridge between meso- and metathorax. I: ventro-lateral margin of mesophragma. Original end divides into two branches, bent postero-laterad.

D2: O: suspended beneath mesophragma. I: suspended above mesofurca. Broad medially and narrowing towards both ends, bent ventro-laterad.

D4: Approximate elongated triangle, narrowing towards mesophragma, straight.

D6: O: ventro-lateral margin of mesophragma. I: suspended above mesofurca. Broad medially and narrowing towards both ends, bent postero-laterad.

D8: Absent.

D10: O: ventro-lateral margin of mesophragma. I: dorso-lateral area of mesofurcal arm. Approximate elongated triangle, narrowing towards mesophragma, bent postero-proximad.

D12: Elongated trapezoid, original end broader than insertional end, straight.

- *IItpm4 M. mesonoto-pleuralis anterior*

D1: Absent.

D2: O: suspended close to mesopleural ridge. I: suspended beneath mesophragma. Broad medially and narrowing towards both ends, straight.

D4: O: antero-lateral area of mesonotum. I: ventral portion of mesopleural ridge. Parallelogram, bent proximad.

D6: Absent.

D8: O: antero-lateral area of mesonotum. I: dorso-median area of mesopleuron. Elongated triangle, narrowing towards mesopleuron, straight.

D10 and 12: Absent.

- *IItpm6 M. mesonoto-pleuralis posterior*

D1: O: central area of mesanepisternum. I: postero-lateral area of mesonotum. Broad medially and narrowing towards both ends, slightly bent postero-proximad.

D2, 4, and 6: Absent.

D8: O: dorso-median area of mesopleuron. I: postero-lateral area of mesonotum. Straight.

D10: O: dorsal portion of mesopleural ridge. I: postero-lateral area of mesonotum. Elongated triangle, narrowing towards the mesopleural ridge, slightly bent ventro-proximad.

D12: Almost the same as that on D10.

- *IItpm7 M. mesanepisterno-axillaris*

D1, 2, 4, 6, and 8: Absent.

D10: O: dorso-median area of mesanepisternum. I: postero-lateral area of mesonotum. Elongated triangle, narrowing towards mesonotum, straight.

D12: Absent.

- *IItpm10 M. mesepimeron-subalaris*

D1: O: postero-median area of mesepimeron. I: suspended beneath mesonotum. Broad medially and narrowing towards both ends, bent proximad.

D2: Absent.

D4: O: dorsal portion of mesopleural ridge. I: postero-lateral area of mesonotum. Bent ventro-proximad.

D6: O: postero-dorsal area of mesepimeron. I: postero-lateral area of mesonotum. Straight.

D8: Absent.

D10: O: dorsal area of the posterior face of the mesopleural ridge. I: postero-lateral area of mesonotum. Flat triangle, narrowing towards the mesonotum, straight.

D12: O: postero-median area of mesepimeron. I: postero-lateral area of mesonotum. Approximate parallelogram, straight.

- *IIspm2 M. mesofurca-pleuralis*

D1, 2, and 4: Absent.

D6: O: median portion of the mesopleural ridge. I: postero-proximal margin of mesocoxal rim. Broad medially and narrowing towards both ends, slightly bent antero-proximad.

D8: Absent.

D10: O: suspended close to mesepimeron. I: postero-lateral area of mesofurcal arm. Approximate parallelogram, bent ventro-proximad.

D12: Absent.

- *IIspm6 M. mesofurca-metaepisternalis*

D1: O: lateral portion of the posterior ridge of mesoventrite. I: median portion of the intersegmental ridge between meso- and metapleuron. Broad medially and narrowing towards both ends, bent dorso-proximad.

D2: O: ventral portion of intersegmental ridge between meso- and metapleuron. I: dorsal portion of intersegmental ridge between meso- and metapleuron. Bent ventro-proximad.

D4: O: suspended close to mesofurca. I: suspended close to the metabasalar sclerite. Straight.

D6: O: suspended close to mesocoxa. I: postero-ventral margin of metabasalar sclerite.

D8, 10, and 12: Absent.

- *IIpcm2 M. mesobasalare-trochantinalis*

D1: O: antero-dorsal area of mesanepisternum. I: lateral portion of the posterior prosternal ridge. Broad medially and narrowing towards both ends, bent postero-laterad.

D2 and 4: Absent.

D6: O: antero-dorsal area of mesanepisternum. I: postero-ventral area of mesepimeron. Elongated rectangle, bent postero-proximad.

D8: Broad medially and narrowing towards both ends, straight.

D10: O: antero-dorsal area of mesanepisternum. I: mesotrochanter.

D12: O: dorsal portion of mesopleural ridge. I: mesotrochanter.

- *IIpcm4 M. mesanepisterno-coxalis posterior*

D1: Absent.

D2: O: ventro-proximal area of process of the mesopleural ridge. I: suspended above mesocoxa. Broad medially and narrowing towards both ends, straight.

D4: O: ventral margin of process of mesopleural ridge. I lateral margin of mesocoxal rim. Slightly bent postero-proximad.

D6: O: dorsal portion of mesopleural ridge. I: lateral margin of mesocoxal rim. Straight.

D8: Curved.

D10 and 12: Absent.

- *IIpcm6 M. mesopleura-trochanteralis*

D1, 2, 4, 6, and 8: Absent.

D10: O: dorsal area of anterior face of the mesopleural ridge. I: mesotrochanter. Broad medially and narrowing towards both ends, straight.

D12: Almost the same as that on D10.

- *IIvlm3 M. mesofurca-metafurcalis*

D1: Two bundles, with the dorsal bundle broader than the ventral bundle. O: postero-dorsal area of mesocoxal posterior ridge. I: anterior margin of the metacoxal rim. Dorsal bundle: elongated trapezoid, original end narrower than insertional end, straight. Ventral bundle: elongated trapezoid, original end broader than insertional end, bent dorso-posterad.

D2: O: postero-lateral area of mesofurcal arm. I: anterior corner of metafurcal arm. Original end divided into three branches, slightly bent dorso-proximad.

D4: O: suspended behind mesofurcal arm. I: suspended in front of metafurcal arm. Elongated triangle, narrowing towards metafurca, straight.

D6: Broad medially and narrowing towards both ends, straight.

D8: Absent.

D10: O: postero-median margin of mesofurcal arm. I: antero-dorsal area of metafurcal arm. Elongated triangle, narrowing towards metafurca, curved.

D12: Almost the same as that on D10.

- *IIscm1 M. mesofurca-coxalis anterior*

D1, 2, 4, 6, and 8: Absent.

D10: O: ventral margin of mesofurcal arm. I: antero-proximal area of mesocoxal ridge. Triangle, narrowing towards mesocoxa, slightly bent ventro-proximad.

D12: O: antero-proximal margin of mesofurcal arm. I: anterior margin of mesocoxal rim. Broad medially and narrowing towards both ends, straight.

- *IIscm2 M. mesofurca-coxalis posterior*

D1, 2, 4, 6, and 8: Absent.

D10: O: ventro-proximal area of mesofurcal arm. I: posterior margin of mesocxal rim. Triangle narrowing towards the mesocoxa, straight.

D12: Almost the same as that on D10.

#### 3.2.3. Metathoracic Muscles

- *IIIdlm1 M. mesophragma-metaphragmalis*

D1: Two bundles, the proximal one longer than the distal one. O: ventro-median area of the posterior face of mesophragma. I: dorso-lateral area of the anterior face of metaphragma. Broad medially and narrowing towards both ends, curved.

D2: O: dorso-median area of posterior face of mesophragma. I: dorso-median area of the anterior face of metaphragma. Elongated rectangle, slightly bent antero-ventrad.

D4: O: ventro-lateral area of the posterior face of mesophragma. I: dorso-median area of the anterior face of metaphragma. Broad medially and narrowing towards both ends, straight.

D6: O: dorso-median area of posterior face of mesophragma. I: suspended beneath the metanotum. Elongated rectangle, bent ventro-anterad.

D8: O: dorso-lateral area of the posterior face of mesophragma. I: dorso-median area of the anterior face of metaphragma. Elongated triangle, narrowing towards metaphragma, straight.

D10: O: dorso-lateral area of the posterior face of mesophragma. I: dorso-median area of anterior face of metaphragma, Elongated rectangle, straight.

D12: Almost the same as that on D10.

- *IIIdlm2 M. metanoto-phragmalis*

D1: O: meso-lateral area of metanotum. I: latero-median area of the anterior face of metaphragma. Broad medially and narrowing towards both ends, bent antero-ventrad.

D2: Almost the same as that on D1.

D4: O: meso-lateral area of metanotum. I: ventro-lateral margin of metaphragma. Elongated triangle, narrowing towards the metanotum, straight.

D6: Broad medially and narrowing towards both ends, slightly bent ventro-posterad.

D8: Elongated triangle, narrowing towards the metanotum, straight.

D10: Parallelogram, bent laterad.

D12: Approximate elongated triangle, narrowing towards the metanotum, straight.

- *IIIdvm1 M. metanoto-sternalis*

D1: O: antero-lateral area of metanotum. I: antero-median area of metaventrite. Broad medially and narrowing towards both ends, curved.

D2: O: suspended beneath metanotum. I: antero-median area of metaventrite. Slightly bent postero-laterad.

D4: Elongated triangle, narrowing towards the metanotum, bent laterad.

D6: O: antero-lateral area of metanotum. I: antero-median area of metaventrite. Broad medially and narrowing towards both ends, bent laterad.

D8: Elongated trapezoid, original end narrower than insertional end, bent postero-dorsad.

D10: Elongated rectangle, bent postero-dorsad.

D12: Almost the same as that on D10.

- *IIIdvm2 M. metanoto-trochantinalis anterior*

D1: O: antero-lateral area of metanotum. I: antero-median area of metaventrite. Elongated rectangle, bent antero-ventrad.

D2: Broad medially and narrowing towards both ends, slightly bent postero-laterad.

D4: O: antero-lateral area of metanotum. I: suspended above the metaventrite. Broad medially and narrowing towards both ends, straight.

D6: Elongated triangle, narrowing towards the metaventrite, curved.

D8: O: antero-lateral area of metanotum. I: postero-median area of metaventrite. Elongated triangle, narrowing towards the metaventrite, straight.

D10: Approximate elongated trapezoid, original end narrower than insertional end, slightly bent postero-dorsad.

D12: Approximate elongated rectangle, curved.

- *IIIdvm4 M. metanoto-coxalis anterior*

D1: Absent.

D2: O: antero-lateral area of metanotum. I: antero-lateral margin of metacoxal rim. Broad medially and narrowing towards both ends, straight.

D4: O: meso-lateral area of metanotum. I: antero-lateral margin of metacoxal rim.

D6: Absent.

D8: O: antero-lateral area of metanotum. I: antero-lateral margin of metacoxal rim. Elongated triangle, narrowing towards the metanotum, straight.

D10: Almost the same as that on D8.

D12: O: meso-lateral area of metanotum. I: antero-lateral margin of metacoxal rim. Elongated triangle, narrowing towards the metacoxa, straight.

- *IIIdvm5 M. metanoto-coxalis posterior*

D1: O: meso-lateral area of the metanotum. I: antero-lateral margin of metacoxal rim. Broad medially and narrowing towards both ends, bent antero-ventrad.

D2: Slightly bent dorso-posterad.

D4: O: postero-lateral area of metanotum. I: antero-lateral margin of metacoxal rim. Slightly bent proximad.

D6: Bent anterad.

D8: O: meso-lateral area of metanotum. I: suspended in front of the metacoxa. Elongated triangle, narrowing towards the metacoxal, slightly bent laterad.

D10: Broad medially and narrowing towards both ends, curved.

D12: O: postero-lateral area of metanotum. I: antero-lateral margin of metacoxal rim. Approximate parallelogram, insertional end expanded, slightly bent proximad.

- *IIIdvm6 M. metacoxal-subalaris*

D1 and 2: Absent.

D4: O: antero-lateral margin of metacoxal rim. I: postero-lateral area of metanotum. Approximate parallelogram, straight.

D6: Absent.

D8: Elongated rectangle, curved.

D10: O: antero-lateral margin of metacoxal rim. I: meso-lateral area of metanotum. Approximate parallelogram, narrowing towards the metanotum, curved.

D12: O: antero-lateral margin of metacoxal rim. I: postero-lateral area of metanotum. Broad medially and narrowing towards both ends, slightly bent postero-dorsad.

- *IIIdvm8 M. metafurca-phragmalis*

D1: O: ventro-proximal area of metafurcal arm. I: ventro-lateral margin of metaphragma. Approximate elongated rectangle, curved.

D2: O: dorso-lateral area of metafurcal arm. I: ventro-lateral margin of metaphragma. Approximate elongated triangle, narrowing towards the metaphragma, curved.

D4: Broad medially and narrowing towards both ends, slightly bent antero-laterad.

D6: Elongated triangle, narrowing towards the metaphragma, bent laterad.

D8: Elongated triangle, narrowing towards metafurca, straight.

D10: Elongated triangle, narrowing towards the metaphragma, slightly bent antero-ventrad.

D12: Approximate elongated triangle, narrowing towards the metaphragma, straight.

- *IIItpm2 M. metapleural-praealaris*

D1: Absent.

D2: O: antero-dorsal area of metapleuron. I: suspended beneath the metanotum. Broad medially and narrowing towards both ends, straight.

D4: O: antero-dorsal area of metapleuron. I: antero-lateral area of metanotum. Approximate rectangle, straight.

D6: Elongated triangle, narrowing towards metapleuron, straight.

D8 and 10: Almost the same as that on D6.

D12: Approximate rectangle, straight.

- *IIItpm3 M. metanoto-basalaris*

D1: Absent.

D2: O: suspended beneath metanotum. I: antero-median area of metapleuron. Broad medially and narrowing towards both ends, slightly bent postero-proximad.

D4: O: antero-lateral area of metanotum. I: dorso-posterior margin of metabaslare. Broad medially and narrowing towards both ends, straight.

D6 and 8: Absent.

D10: O: antero-lateral area of metanotum. I: suspended close to metabasalar sclerite. Approximate rectangle, straight.

D12: O: antero-lateral area of metanotum. I: dorso-proximal area of metabasalar sclerite. Trapezoid, original end narrower than insertional end, straight.

- *IIItpm5 M. metanoto-pleuralis medialis*

D1, 2, 4, 6, 8, and 10: Absent.

D12: O: dorso-distal area of metapleural ridge. I: meso-lateral margin of metanotum. Triangle, narrowing towards the metanotum, straight.

- *IIItpm6 M. metanoto-pleuralis posterior*

D1: Absent.

D2: O: postero-median area of metapleuron. I: postero-lateral margin of metanotum. Broad medially and narrowing towards both ends, straight.

D4: Absent.

D6: O: suspended close to metapleuron. I: meso-lateral margin of metanotum. Elongated triangle, narrowing towards the metapleuron, curved.

D8: Absent.

D10: O: antero-median area of metapleuron. I: meso-lateral margin of metanotum. Elongated triangle, narrowing towards the metanotum, straight.

D12: O: dorso-median area of metapleural ridge. I: postero-lateral margin of metanotum. Elongated triangle, narrowing towards the metanotum, slightly bent proximad.

- *IIItpm7 M. metanepisterno-axillaris*

D1, 2, and 4: Absent.

D6: O: dorso-median area of metapleuron. I: postero-lateral margin of metanotum. Approximate elongated trapezoid, original end broader than insertional end, bent antero-ventrad.

D8: Broad medially and narrowing towards both ends, straight.

D10: O: dorso-anterior area of metapleuron. I: meso-lateral margin of metanotum. Approximate trapezoid, original end broader than insertional end, straight.

D12: O: dorso-proximal area of metapleural ridge. I: antero-lateral margin of metanotum. Approximate elongated triangle, narrowing towards the metanotum, insertional end expands, straight.

- *IIIspm1 M. metapleural-sternalis*

D1: Absent.

D2: O: metabasalar sclerite. I: latero-median area of metaventrite. Approximate elongated triangle, narrowing towards the metabasalar sclerite, slightly bent postero-dorsad.

D4: Almost the same as that on D2.

D6: Elongated triangle, narrowing towards the metabasalar sclerite, slightly bent antero-ventrad.

D8: Slightly bent postero-dorsad.

D10 and 12: Almost the same as that on D8.

- *IIIpcm2 M. metabasalar sclerite-trochantinalis*

D1: Absent.

D2: O: antero-dorsal area of metapleuron. I: antero-lateral margin of metacoxal rim. Broad medially and narrowing towards both ends, slightly bent postero-dorsad.

D4: Elongated triangle, narrowing towards the metapleuron, straight.

D6: Almost the same as that on D4.

D8 and 10: Absent.

D12: O: ventro-lateral area of metapleural ridge. I: extends towards the metacoxa. Triangle, narrowing towards the metacoxal, slightly bent antero-ventrad.

- *IIIpcm3 M. metanepisterno-coxalis anterior*

D1: Absent.

D2: O: antero-dorsal area of metapleuron. I: antero-lateral margin of metacoxal rim. Approximate elongated triangle, narrowing towards the metapleuron, straight.

D4, 6, 8, 10, and 12: Absent.

- *IIIpcm4 M. metanepisterno-coxalis posterior*

D1 and 2: Absent.

D4: O: central area of metapleuron. I: antero-lateral margin of metacoxal rim. Broad medially and narrowing towards both ends, bent postero-dorsad.

D6 and 8: Absent.

D10: Elongated triangle, narrowing towards the metacoxa, bent postero-dorsad.

D12: O: ventro-median area of metapleural ridge. I: antero-lateral margin of metacoxal rim. Approximate elongated triangle, narrowing towards the metapleural ridge, straight.

- *IIIscm1 M. metafurca-coxalis anterior*

D1 and 2: Absent.

D4: O: suspended beneath metafurcal arm. I: extends towards the metacoxa. Elongated triangle, narrowing towards metacoxal, straight.

D6: Absent.

D8: O: ventro-proximal area of metafurcal arm. I: antero-proximal margin of metacoxal rim.

D10: O: ventro-lateral area of metafurcal stem. I: antero-proximal margin of metacoxal rim.

D12: Almost the same as that on D10.

- *IIIscm2 M. metafurca-coxalis posterior*

D1, 2, 4, 6, and 8: Absent.

D10: O: posterior margin of metafurcal arm. I: anterior margin of the metacoxal rim. Broad and flat, straight.

D12: O: postero-lateral margin of metafurcal arm. I: antero-lateral margin of metacoxal rim. Trapezoid, original end broader than insertional end, straight.

- *IIIscm3 M. metafurca-coxalis medialis*

D1: Absent.

D2: O: ventro-lateral area of metafurcal arm. I: extends towards the metacoxal. Broad medially and narrowing towards both ends, slightly bent antero-dorsad.

D4: O: postero-proximal margin of metafurcal arm. I: antero-proximal margin of metacoxal rim. Elongated triangle, narrowing towards metafurca, straight.

D6: Absent.

D8: Parallelogram, straight.

D10: Broad and flat, straight.

D12: Almost the same as that on D8.

- *IIIscm4 M. metafurca-coxalis lateralis*

D1: O: suspended above mesoventrite. I: antero-proximal margin of metacoxa. Broad medially and narrowing towards both ends, straight.

D2: O: dorso-lateral area of metafurcal arm. I: antero-lateral margin of metacoxa. Elongated triangle, narrowing towards the metacoxa, straight.

D4: O: ventro-anterior area of metafurcal arm. I: antero-lateral margin of metacoxa. Elongated triangle, narrowing towards metafurca, slightly bent dorsad.

D6: Absent.

D8: O: ventro-proximal area of metafurcal arm. I: extends towards the metacoxa. Elongated triangle, narrowing towards the metacoxa.

D10: O: ventral area of metafurcal arm. I: antero-lateral margin of metacoxal rim. Approximate triangle, narrowing towards the metacoxa, straight.

D12: Almost the same as that on D10.

- *IIIscm6 M. metafurca-trochanteralis*

D1 and 2: Absent.

D4: O: ventro-lateral area of metafurcal arm. I: suspended above the metacoxa. Broad medially and narrowing towards both ends, straight.

D6 and 8: Absent.

D10: O: ventro-lateral area of metafurcal arm. I: metatrochanter. Elongated triangle, narrowing towards metatrochanter, slightly curved.

D12: Almost the same as that on D10.

### 3.3. Digestive System and Nervous System

Overall, the gut and nerve cord show little change during pupal development. The thick gut (gut: Figure 2(D1-4), Figure 3(D2-4), Figure 4(D4-4), Figure 5(D6-4), Figure 6(D8-4), Figure 8(D10-4), and Figure 9(D12-4)) is located in the center of the thoracic cavity. Throughout all pupal developmental stages, the gut shows a gradual widening from anterior to posterior. In the prothoracic region, the gut is very slender on D1 (gut: Figure 2(D1-4)) and begins to thicken from D2 onward. The nerve cord lies close above the furcae and contains an enlarged ganglion in each of the pro-, meso-, and metathoracic segments (g1–3: Figure 2(D1-4), Figure 3(D2-4), Figure 4(D4-4), Figure 5(D6-4), Figure 6(D8-4), Figure 8(D10-4), and Figure 9(D12-4)). The condition of the metathoracic ganglion on D4 is most likely attributable to specimen quality or to limitations in the micro-CT scan (Figure 4(D4-4)).

## 4. Discussion

From the dorsal view, the relative length of the mesonotum gradually decreases during pupal development. From D1 to D6, the mesonotum is about half the length of the pronotum or metanotum, but after D8 it is reduced to roughly 1/3. Thoracic length, width, and height do not all increase progressively during development, which might either reflect the inherent complexity of pupal growth or arise from variation among individuals (Table 1). From D2 to D8, a membrane extends between the occiput and the metaphragma beneath the thoracic notum, serving as the attachment site for the notal muscles. During individual development, the main deformations of the endoskeleton occur in the phragma, crytopleuron, pleural ridge, and furca. The profurca first appears on D2, the crytopleuron on D10, the metabasalar sclerite on D2, and the metapleural ridge on D12. These endoskeletal structures serve as attachment sites for some muscles. Consequently, the origin or insertion points of the associated muscles change accordingly. On D10 and D12, Itpm3 and 4, and Ipcm5 and 8 attach to the crytopleuron, whereas in earlier stages their ends are either connected to the pronotum or suspended nearly. Ivlm3 and 7, which should attach to the profurcal arm, are instead connected to the prosternal posterior ridge on D1. From D2 onward, IIspm6 and IIItpm3 attach to the metabasalar sclerite, whereas on D1 they are connected either to the intersegmental ridge between the meso- and metapleuron or directly to the metapleuron. Prior to D12, IIItpm6 and 7, and IIIpcm2 and 4 attach directly to the metapleuron, and only at D12 do they connect to the metapleural ridge. Otherwise, Idvm10 and 12, Iscm4, IIIspm1, and IIItpm5 emerge only after the formation of the profurca, metabasalar sclerite, or the metapleural ridge.

**Figure 10:**
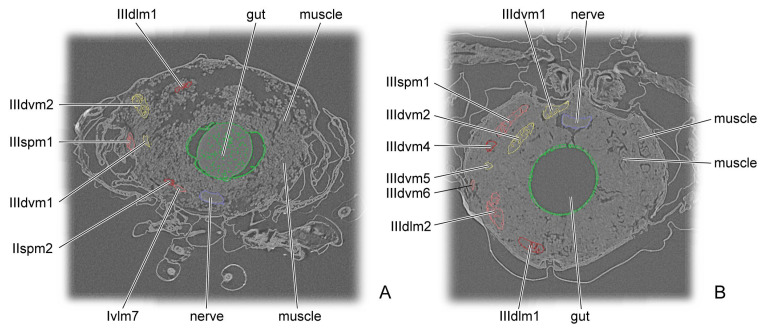
Cross sections in micro-CT scans of pupal *Asiophrida xanthospilota*. (**A**) D6; (**B**) D8. Muscles are marked in red, pink, or yellow; gut in green; and nerves in dark blue.

Across all pupal stages, the micro-CT slices reveal a thoracic cavity filled with high-density stroma, whose brightness is nearly indistinguishable from that of muscle, thereby greatly hindering muscle identification (Figure 10). The variation in muscular number during pupal development appears highly irregular. Many muscles are present in both the early and late stages but disappear during the middle stages, a pattern that might be influenced by the quality of the micro-CT results. Surprisingly, most muscles are already present at D1, with the exception of some metathoracic muscles, indicating that their formation begins in the late larval stage. The prothorax contains 17 muscles, and the mesothorax contains 11 muscles. From D2 to D8, the prothorax has only 13 to 14 muscles, whereas the mesothorax has 7 to 10 muscles (Table 2). By comparison, the flight-form *Callosobruchus maculatus* develops only seven muscles in the metathorax during the initial pupal stage [35]. *Asiophrida xanthospilota* exhibits excellent jumping ability. Whether that early development of the thoracic muscles during the pupal stage is related to this ability remains uncertain. Although jumping in flea beetles primarily relies on the hind leg, it probably also represents a whole-body exertion that requires coordination of the entire locomotor system. In *A. xanthospilota*, the principal indirect flight muscles—IIIspm1, and IIIpcm2 and 4—are absent on D1. In contrast, in the flight form *C. maculatus*, the indirect flight muscles IIIspm1, IIIpcm3, and 4 are already established in the prepupal stage [35]. This pattern probably suggests that, because *A. xanthospilota* relies more on hind-leg jumping, developmental energy is preferentially allocated to the leg muscles rather than to the thoracic flight muscles during early pupal development. Some muscles emerge during the early or middle stages of pupal development but disappear in the later stages, including Idlm6, Idvm8, 14, and 15, Iscm4, IItpm4 and 7, IIspm2, IIpcm4, and IIIpcm3. They might provide structural support at a time when sclerotization is still relatively weak.

For each developmental stage, we described in detail the origin and insertion of every muscle, along with its planar shape, direction of curvature, and the changes occurring during development. We also calculated the absolute and relative volumes of each muscle. Most muscles undergo considerable morphological variation, making it difficult to discern consistent patterns. Muscular attachment sites shift on the endoskeleton, with muscles exhibiting planar shapes such as triangular, trapezoidal, or rectangular. Their fibers might be straight, twisted, or curved in a particular direction. Some muscle ends appear to be suspended near the endoskeleton, likely supported by the thoracic stroma or the other tissues [39]. Comparable phenomena have also been reported in *Chrysopa pollen*, *Drosophila melanogaster*, and *Callosobruchus maculatus* [29,30,35]. The pupa on D4 reach their greatest thoracic length, width, and height; however, the absolute volumes of most muscles are smaller than in the preceding and subsequent stages, resulting in reduced relative volumes (Table 3 and Table 4). This result is likely due to experimental error or to the high accumulation of stroma within the thoracic cavity, which compresses the muscles on D4.

The morphology of the gut and nerve in the thorax remains almost identical to that of *Tenebrio molitor*, *Tribolium castaneum*, and *Harmonia axyridis* [33,34].

## Figures and Tables

**Table 1:** Thoracic length, width, and height (mm) of the pupae at each pupal developmental stage.

	D1	D2	D4	D6	D8	D10	D12
Length	2.07	3.03	3.46	3.23	2.32	2.68	2.95
	2.05	2.92	3.35	3.17	2.33	2.66	3.00
	2.06	2.94	3.39	3.13	2.34	2.63	2.96
Width	1.83	2.21	2.50	2.42	1.59	1.97	1.80
	1.79	2.34	2.54	2.30	1.59	2.04	1.75
	1.84	2.26	2.58	2.33	1.56	2.09	1.81
Heigh	1.71	2.13	2.31	2.02	1.77	1.55	1.99
	1.66	2.21	2.35	1.91	1.66	1.55	1.86
	1.67	2.26	2.31	1.92	1.73	1.56	1.94
Product	6.48	14.3	20.0	15.8	6.53	8.18	10.6
	6.09	15.1	20.0	13.9	6.15	8.41	9.77
	6.33	15.0	20.2	14.0	6.32	8.57	10.4
Mean of products	6.30	14.8	20.1	14.6	6.33	8.39	10.3

**Table 2:** Thoracic muscles in each pupal developmental stage. Present is denoted with “+” and green color, and absence is denoted with “−” and orange color. At each stage, the total numbers of pro-, meso-, and metathoracic muscles are listed in the row labeled “Sum”, while the overall number of identified muscles is provided in parentheses next to “Sum”.

	D1	D2	D4	D6	D8	D10	D12
Prothorax							
Idlm1	+	+	+	+	+	+	+
Idlm2	−	−	−	+	+	+	+
Idlm3	+	−	−	+	−	−	+
Idlm4	+	−	+	+	+	+	+
Idlm5	+	+	+	+	+	+	+
Idlm6	+	+	+	−	+	−	−
Idvm1	+	+	−	+	−	+	+
Idvm5	+	+	+	+	+	+	+
Idvm7	−	−	+	+	−	+	+
Idvm8	+	+	+	−	+	+	−
Idvm9	+	−	−	−	−	−	−
Idvm10	−	+	+	+	+	+	+
Idvm12	−	−	−	−	−	+	+
Idvm14	−	−	+	−	−	−	−
Idvm15	−	−	+	−	−	−	−
Idvm16	+	+	+	+	+	+	+
Idvm17	+	+	+	+	+	+	+
Itpm3	+	−	−	−	+	+	+
Itpm4	+	+	−	−	−	+	+
Itpm6	+	−	−	−	−	−	+
Ipcm5	+	−	−	−	−	+	+
Ipcm8	−	−	−	+	−	+	+
Ivlm3	+	+	−	+	+	+	+
Ivlm7	+	+	+	+	+	+	+
Iscm4	−	+	−	−	−	−	−
Sum (25)	17	13	13	14	13	18	19
Mesothorax							
IIdlm1	+	+	+	−	+	+	+
IIdlm2	+	+	+	−	−	+	+
IIdvm2	+	−	+	−	−	+	+
IIdvm4	+	+	+	+	+	+	+
IIdvm5	+	−	−	−	+	−	+
IIdvm8	+	+	+	+	−	+	+
IItpm4	−	+	+	−	+	−	−
Itpm6	+	−	−	−	+	+	+
IItpm7	−	−	−	−	−	+	−
IItpm10	+	−	+	+	−	+	+
IIspm2	−	−	−	+	−	+	−
IIspm6	+	+	+	+	−	−	−
IIpcm2	+	−	−	+	+	+	+
IIpcm4	−	+	+	+	+	−	−
IIpcm6	−	−	−	−	−	+	+
IIvlm3	+	+	+	+	−	+	+
IIscm1	−	−	−	−	−	+	+
IIscm2	−	−	−	−	−	+	+
Sum (18)	11	8	10	8	7	14	13
Metathorax							
IIIdlm1	+	+	+	+	+	+	+
IIIdlm2	+	+	+	+	+	+	+
IIIdvm1	+	+	+	+	+	+	+
IIIdvm2	+	+	+	+	+	+	+
IIIdvm4	−	+	+	−	+	+	+
IIIdvm5	+	+	+	+	+	+	+
IIIdvm6	−	−	+	−	+	+	+
IIIdvm8	+	+	+	+	+	+	+
IIItpm2	−	+	+	+	+	+	+
IIItpm3	−	+	+	−	−	+	+
IIItpm5	−	−	−	−	−	−	+
IIItpm6	−	+	−	+	−	+	+
IIItpm7	−	−	−	+	+	+	+
IIIspm1	−	+	+	+	+	+	+
IIIpcm2	−	+	+	+	−	−	+
IIIpcm3	−	+	−	−	−	−	−
IIIpcm4	−	−	+	−	−	+	+
IIIscm1	−	−	+	−	+	+	+
IIIscm2	−	−	−	−	−	+	+
IIIscm3	−	+	+	−	+	+	+
IIIscm4	+	+	+	−	+	+	+
IIIscm6	−	−	+	−	−	+	+
Sum (22)	7	15	17	11	14	19	21
Total number of thoracic muscles							
65	35	36	40	33	34	51	53

**Table 3:** The absolute volume of the thoracic muscles (µm^3^/10^5^) at each pupal developmental stage. The absolute volume larger than that at the last developmental stage is denoted in blue, while the smaller volume is denoted in yellow. Absence is denoted with “−”. On D10, the merged IIIscm2 and 3 are denoted in orange and excluded from the comparison.

	D1	D2	D4	D6	D8	D10	D12
Prothorax							
Idlm1	4.355	4.739	12.98	33.82	12.77	18.05	24.06
Idlm2	−	−	−	4.919	8.458	4.349	19.30
Idlm3	2.150	−	−	7.747	−	−	4.972
Idlm4	6.661	−	1.135	8.356	5.321	15.64	9.480
Idlm5	10.06	17.09	8.952	12.76	13.37	55.76	63.00
Idlm6	16.20	25.65	8.939	−	11.35	−	−
Idvm1	7.646	5.957	−	6.727	−	15.84	25.34
Idvm5	12.23	8.209	4.142	9.838	11.78	12.36	22.98
Idvm7	−	−	2.715	9.282	−	37.12	44.19
Idvm8	12.71	14.61	9.162	−	1.576	2.059	−
Idvm9	0.9397	−	−	−	−	−	−
Idvm10	−	7.095	4.265	7.347	5.944	14.12	18.29
Idvm12	−	−	−	−	−	21.79	4.886
Idvm14	−	−	3.558	−	−	−	−
Idvm15	−	−	9.892	−	−	−	−
Idvm16	5.248	20.91	1.858	8.031	23.87	14.44	46.74
Idvm17	5.116	15.43	7.365	20.97	25.16	7.789	40.07
Itpm3	1.219	−	−	−	1.340	3.250	1.332
Itpm4	10.34	3.067	−	−	−	8.961	4.787
Itpm6	1.866	−	−	−	−	−	18.57
Ipcm5	1.986	−	−	−	−	12.48	6.129
Ipcm8	−	−	−	18.08	−	22.90	32.22
Ivlm3	25.67	35.60	−	5.646	5.404	29.12	31.79
Ivlm7	44.66	12.90	9.056	15.88	7.227	32.16	49.69
Iscm4	−	4.319	−	−	−	−	−
Mesothorax							
IIdlm1	8.394	4.847	3.422	−	10.31	47.91	50.54
IIdlm2	0.5124	5.537	2.662	−	−	8.570	19.50
IIdvm2	10.02	−	1.571	−	−	23.56	8.732
IIdvm4	6.964	11.72	5.748	11.07	12.72	24.41	35.56
IIdvm5	14.97	−	−	−	3.913	−	8.828
IIdvm8	8.294	7.860	2.609	6.572	−	6.094	7.309
IItpm4	−	4.681	1.725	−	4.531	−	−
Itpm6	3.307	−	−	−	3.284	1.244	0.9824
IItpm7	−	−	−	−	−	5.512	−
IItpm10	1.998	−	1.263	2.876	−	6.477	10.53
IIspm2	−	−	−	7.861	−	7.056	−
IIspm6	3.758	10.73	1.996	4.395	−	−	−
IIpcm2	6.280	−	−	12.29	1.245	12.41	20.84
IIpcm4	−	1.055	4.753	6.886	3.796	−	−
IIpcm6	−	−	−	−	−	11.70	9.947
IIvlm3	64.14	18.43	2.411	0.8039	−	10.66	12.43
IIscm1	−	−	−	−	−	12.98	2.762
IIscm2	−	−	−	−	−	12.62	13.00
Metathorax							
IIIdlm1	12.84	61.92	60.87	61.37	98.42	173.0	252.7
IIIdlm2	11.75	28.74	39.40	50.82	59.92	256.3	238.3
IIIdvm1	32.36	45.54	51.52	34.05	89.32	157.3	198.7
IIIdvm2	15.36	77.63	94.67	145.7	166.5	327.0	483.7
IIIdvm4	−	11.62	6.704	−	38.04	70.03	38.06
IIIdvm5	7.978	8.574	10.50	11.06	10.65	18.25	42.03
IIIdvm6	−	−	1.643	−	14.24	38.87	10.71
IIIdvm8	6.792	19.64	8.207	14.73	23.22	12.57	24.08
IIItpm2	−	6.726	3.808	5.266	6.320	6.202	8.134
IIItpm3	−	4.431	2.120	−	−	10.20	6.736
IIItpm5	−	−	−	−	−	−	2.759
IIItpm6	−	5.960	−	11.77	−	12.71	23.69
IIItpm7	−	−	−	7.150	3.235	13.84	9.411
IIIspm1	−	65.29	72.67	123.5	133.2	313.2	474.6
IIIpcm2	−	16.73	21.97	47.22	−	−	17.46
IIIpcm3	−	6.115	−	−	−	−	−
IIIpcm4	−	−	3.528	−	−	35.51	38.81
IIIscm1	−	−	3.208	−	9.336	22.33	32.23
IIIscm2	−	−	−	−	−	47.24	5.493
IIIscm3	−	5.039	1.336	−	10.15		30.39
IIIscm4	1.507	9.527	5.420	−	2.760	24.44	28.48
IIIscm6	−	−	2.209	−	−	18.61	14.38

**Table 4:** The relative volume of the thoracic muscles (magnified 10^5^ times) at each pupal developmental stage. The relative volume larger than that at the last developmental stage is denoted in blue, while the smaller volume is denoted in yellow. Absence is denoted with “−”. On D10, the merged IIIscm2 and 3 are denoted in orange and excluded from the comparison.

	D1	D2	D4	D6	D8	D10	D12
Prothorax							
Idlm1	6.91	3.20	6.46	23.2	2.02	21.5	23.4
Idlm2	−	−	−	3.37	13.4	5.18	18.7
Idlm3	3.41	−	−	5.31	−	−	4.83
Idlm4	10.6	−	0.565	5.72	8.41	18.6	9.20
Idlm5	16.0	11.5	4.45	8.74	21.1	66.5	61.2
Idlm6	25.7	17.3	4.45	−	17.9	−	−
Idvm1	12.1	4.03	−	4.61	−	18.9	24.6
Idvm5	19.4	5.55	2.06	6.74	18.6	14.7	22.3
Idvm7	−	−	1.35	6.36		44.2	42.9
Idvm8	20.2	9.87	4.56	−	2.49	2.45	−
Idvm9	1.49	−	−	−	−	−	−
Idvm10	−	4.79	2.12	5.03	9.39	16.8	17.8
Idvm12	−	−	−	−	−	26.0	4.74
Idvm14	−	−	1.77	−	−	−	−
Idvm15	−	−	4.92	−	−	−	−
Idvm16	8.33	14.1	0.924	5.50	37.7	17.2	45.4
Idvm17	8.12	10.4	3.66	14.4	39.7	9.28	38.9
Itpm3	1.93	−	−	−	2.12	3.87	1.29
Itpm4	16.4	2.07	−	−	−	10.7	4.65
Itpm6	2.96	−	−	−	−	−	18.0
Ipcm5	3.15	−	−	−	−	14.9	5.95
Ipcm8	−	−	−	12.4	−	27.3	31.3
Ivlm3	40.7	24.1	−	3.87	8.54	34.7	30.9
Ivlm7	70.9	8.72	4.51	10.9	11.4	38.3	48.2
Iscm4	−	2.92	−	−	−	−	−
Mesothorax							
IIdlm1	13.3	3.28	1.70	−	16.3	57.1	49.1
IIdlm2	0.813	3.74	1.32	−	−	10.2	18.9
IIdvm2	15.9	−	0.782	−	−	28.1	8.48
IIdvm4	11.1	7.92	2.86	7.58	20.1	29.1	34.5
IIdvm5	23.8	−	−	−	6.18	−	8.57
IIdvm8	13.2	5.31	1.30	4.50	−	7.26	7.10
IItpm4	−	3.16	0.858	−	7.16	−	−
Itpm6	5.25	−	−	−	5.19	1.48	0.954
IItpm7	−	−	−	−	−	6.60	−
IItpm10	3.17	−	0.628	1.97	−	7.72	10.2
IIspm2	−	−	−	5.38	−	8.41	−
IIspm6	5.97	7.25	0.993	3.01	−	−	−
IIpcm2	9.97	−	−	8.42	1.97	14.8	20.2
IIpcm4	−	0.713	2.36	4.72	6.00	−	−
IIpcm6	−	−	−	−	−	13.9	9.66
IIvlm3	102	12.5	1.20	0.551	−	12.7	12.1
IIscm1	−	−	−	−	−	15.5	2.68
IIscm2	−	−	−	−	−	15.0	12.6
Metathorax							
IIIdlm1	20.4	41.8	30.3	42.0	155	206	245
IIIdlm2	18.7	19.4	19.6	34.8	94.7	305	231
IIIdvm1	51.4	30.8	25.6	23.3	141	187	193
IIIdvm2	24.4	52.5	47.1	99.8	263	390	470
IIIdvm4	−	7.85	3.34	−	60.1	83.5	37.0
IIIdvm5	12.7	5.79	5.22	7.58	16.8	21.8	40.8
IIIdvm6	−	−	0.817	−	22.5	46.3	10.4
IIIdvm8	10.8	13.3	4.08	10.1	36.7	15.0	23.4
IIItpm2	−	4.54	1.89	3.61	9.98	7.39	7.90
IIItpm3	−	2.99	1.05	−	−	12.2	6.54
IIItpm5	−	−	−	−	−	−	2.68
IIItpm6	−	4.03	−	8.06	−	15.1	23.0
IIItpm7	−	−	−	4.90	5.11	16.5	9.14
IIIspm1	−	44.1	36.2	84.6	210	373	461
IIIpcm2	−	11.3	10.9	32.3	−	−	17.0
IIIpcm3	−	4.13	−	−	−	−	−
IIIpcm4	−	−	1.76	−	−	42.3	37.7
IIIscm1	−	−	1.60	−	14.7	26.6	31.3
IIIscm2	−	−	−	−	−	56.3	5.33
IIIscm3	−	3.40	0.665	−	16.0		29.5
IIIscm4	2.39	6.44	2.70	−	4.36	29.1	27.7
IIIscm6	−	−	1.10	−	−	22.2	14.0

## Data Availability

The original contributions presented in this study are included in the article. Further inquiries can be directed to the corresponding authors.
